# Comparing one dose of HPV vaccine in girls aged 9–14 years in Tanzania (DoRIS) with one dose of HPV vaccine in historical cohorts: an immunobridging analysis of a randomised controlled trial

**DOI:** 10.1016/S2214-109X(22)00306-0

**Published:** 2022-09-13

**Authors:** Kathy Baisley, Troy J Kemp, Aimée R Kreimer, Partha Basu, John Changalucha, Allan Hildesheim, Carolina Porras, Hilary Whitworth, Rolando Herrero, Charles J Lacey, John T Schiller, Eric Lucas, Paul Mutani, Joakim Dillner, Jackton Indangasi, Richard Muwonge, Richard J Hayes, Ligia A Pinto, Deborah Watson-Jones

**Affiliations:** a Faculty of Epidemiology and Population Health, London School of Hygiene & Tropical Medicine, London, UK; b Faculty of Infectious and Tropical Diseases, London School of Hygiene & Tropical Medicine, London, UK; c HPV Serology Laboratory, Frederick National Laboratory for Cancer Research, Frederick, MD, USA; d Division of Cancer Epidemiology and Genetics, National Cancer Institute, Bethesda, MD, USA; e Early Detection, Prevention and Infections Branch, International Agency for Research on Cancer, World Health Organization, Lyon, France; f Mwanza Intervention Trials Unit, National Institute for Medical Research, Mwanza, Tanzania; g Agencia Costarricense de Investigaciones Biomedicas (ACIB), Fundacion INCIENSA, San Jose, Costa Rica; h York Biomedical Research Institute and Hull York Medical School, University of York, York, UK; i Karolinska Institute, Stockholm, Sweden

## Abstract

**Background:**

Human papillomavirus (HPV) vaccines are given as a two-dose schedule in children aged 9–14 years, or as three doses in older individuals. We compared antibody responses after one dose of HPV vaccine in the Dose Reduction Immunobridging and Safety Study (DoRIS), a randomised trial of different HPV vaccine schedules in Tanzania, to those from two observational HPV vaccine trials that found high efficacy of one dose up to 11 years against HPV16 and HPV18 (Costa Rica Vaccine Trial [CVT] and Institutional Agency for Research on Cancer [IARC] India trial).

**Methods:**

In this immunobridging analysis of an open-label randomised controlled trial, girls were recruited from 54 government schools in Mwanza, Tanzania, into the DoRIS trial. Girls were eligible if they were aged 9–14 years, healthy, and HIV negative. Participants were randomly assigned (1:1:1:1:1:1), using permutated block sizes of 12, 18, and 24, to one, two, or three doses of the 2-valent vaccine (Cervarix, GSK Biologicals, Rixensart, Belgium) or the 9-valent vaccine (Gardasil 9, Sanofi Pasteur MSD, Lyon, France). For this immunobridging analysis, the primary objective was to compare geometric mean concentrations (GMCs) at 24 months after one dose in the per-protocol population compared with in historical cohorts: the one-dose 2-valent vaccine group in DoRIS was compared with recipients of the 2-valent vaccine Cervarix from CVT and the one-dose 9-valent vaccine group in DoRIS was compared with recipients of the 4-valent vaccine Gardasil (Merck Sharp & Dohme, Whitehouse Station, NJ, USA) from the IARC India trial. Samples were tested together with virus-like particle ELISA for HPV16 and HPV18 IgG antibodies. Non-inferiority of GMC ratios (DoRIS trial *vs* historical cohort) was predefined as when the lower bound of the 95% CI was greater than 0·50. This study is registered with ClinicalTrials.gov, NCT02834637.

**Findings:**

Between Feb 23, 2017, and Jan 6, 2018, we screened 1002 girls for eligibility, of whom 930 were enrolled into DoRIS and 155 each were assigned to one dose, two doses, or three doses of 2-valent vaccine, or one dose, two doses, or three doses of 9-valent vaccine. 154 (99%) participants in the one-dose 2-valent vaccine group (median age 10 years [IQR 9–12]) and 152 (98%) in the one-dose 9-valent vaccine group (median age 10 years [IQR 9–12]) were vaccinated and attended the 24 month visit, and so were included in the analysis. 115 one-dose recipients from the CVT (median age 21 years [19–23]) and 139 one-dose recipients from the IARC India trial (median age 14 years [13–16]) were included in the analysis. At 24 months after vaccination, GMCs for HPV16 IgG antibodies were 22·9 international units (IU) per mL (95% CI 19·9–26·4; n=148) for the DoRIS 2-valent vaccine group versus 17·7 IU/mL (13·9–22·5; n=97) for the CVT (GMC ratio 1·30 [95% CI 1·00–1·68]) and 13·7 IU/mL (11·9–15·8; n=145) for the DoRIS 9-valent vaccine group versus 6·7 IU/mL (5·5–8·2; n=131) for the IARC India trial (GMC ratio 2·05 [1·61–2·61]). GMCs for HPV18 IgG antibodies were 9·9 IU/mL (95% CI 8·5–11·5: n=141) for the DoRIS 2-valent vaccine group versus 8·0 IU/mL (6·4–10·0; n=97) for the CVT trial (GMC ratio 1·23 [95% CI 0·95–1·60]) and 5·7 IU/mL (4·9–6·8; n=136) for the DoRIS 9-valent vaccine group versus 2·2 IU/mL (1·9–2·7; n=129) for the IARC India trial (GMC ratio 2·12 [1·59–2·83]). Non-inferiority of antibody GMCs was met for each vaccine for both HPV16 and HPV18.

**Interpretation:**

One dose of HPV vaccine in young girls might provide sufficient protection against persistent HPV infection. A one-dose schedule would reduce costs, simplify vaccine delivery, and expand access to the vaccine.

**Funding:**

UK Department for International Development/UK Medical Research Council/Wellcome Trust Joint Global Health Trials Scheme, The Bill & Melinda Gates Foundation, and the US National Cancer Institute.

**Translation:**

For the KiSwahili translation of the abstract see Supplementary Materials section.

**Research in context**

**Evidence before this study**

We identified a 2019 review of published reports of the efficacy of single dose HPV vaccination. All studies in the review were observational studies of participants in three large HPV vaccine trials who did not complete their allocated schedules. These included the International Agency for Research on Cancer (IARC) HPV vaccine trial in India, the Costa Rica Vaccine Trial (CVT), and the PATRICIA multicentre trial conducted in 14 countries. HPV16 and HPV18 infection was rare in all vaccinated participants up to 7 years after the first dose and all studies reported comparable efficacy of one, two, and three doses of HPV vaccine against HPV16 and HPV18 infection despite differences in antibody levels between the dose groups. We updated this review by searching the Medline, EMBASE, Global Health Database, and Cochrane Central Register of Controlled Trials databases for publications between Aug 1, 2018, and Dec 10, 2021, using the terms “human papillomavirus” AND “vaccines” AND (“immunogenicity” OR “efficacy/effectiveness”) AND “dosage”. We identified two additional studies that extended the CVT and IARC India studies, which found that vaccine efficacy against HPV16 and HPV18 infection endpoints was similar between participants who received one, two, or three doses, and antibody responses remained stable over 11 years for CVT and 9 years for IARC India. Additionally, we identified the first randomised controlled trial of single dose HPV vaccine efficacy, the KEN SHE trial, in girls and women aged 15–20 years in Kenya, which found 97·5% vaccine efficacy for one dose of HPV vaccine compared with a control vaccine at 18 months. However, there is still a paucity of efficacy data from girls in the target age for vaccination (9–14 years).

**Added value of this study**

The Dose Reduction Immunobridging and Safety Study (DoRIS) trial in Tanzanian girls is the first randomised clinical trial to our knowledge to assess the safety and immune responses of a single dose of HPV vaccine compared with two and three doses in girls in the target age for vaccination (9–14 years). Here we present an immunobridging study comparing single-dose vaccine immunogenicity data from the DoRIS trial with historical immunogenicity and efficacy against persistent HPV16 and HPV18 infection data derived from single-dose recipients from two previous, large HPV vaccine clinical trials (CVT and IARC India). We found that HPV16 and HPV18 antibody concentrations and seropositivity at 24 months after one dose in young girls in Tanzania were non-inferior to those in adult women (aged 18–25 years) who received one dose in the CVT or girls (aged 10–18 years) who received one dose in the IARC India trial.

**Implications of all the available evidence**

One dose of HPV vaccine induces antibody responses that are comparable in different geographies and contexts, and a single dose is likely to be effective against persistent HPV16 and HPV18 infection and associated disease. A single dose HPV vaccine schedule could substantially reduce the costs of vaccine purchase and delivery, alleviate vaccine supply constraints, and expand access to the vaccine in the countries that need it most.

## Introduction

The elimination of cervical cancer, caused by human papillomavirus (HPV) infection, is high on the public health agenda following WHO's 2020 global call for action.^1^ Sub-Saharan Africa has the highest cervical cancer incidence and mortality rates globally, and access to screening is often restricted or absent.^2^ Prophylactic HPV virus-like particle (VLP) vaccines are safe and effective in preventing cervical HPV infection and its sequelae. However, estimated global HPV vaccine coverage among girls aged 9–14 years in 2019 was only 15% for full vaccination and 7% in Gavi, the Vaccine Alliance, eligible countries.^3^

Four licensed HPV vaccines are available: the two 2-valent vaccines (Cervarix [GSK Biologicals, Rixensart, Belgium] and Cecolin [Xiamen Innovax Biotech, Xiamen, China]) that target HPV16 and HPV18; the 4-valent vaccine (Gardasil [Merck Sharp & Dohme, Whitehouse Station, NJ, USA]) that targets HPV 6, HPV11, HPV16, and HPV18; and the 9-valent vaccine (Gardasil-9 [Sanofi Pasteur MSD, Lyon, France]) that targets nine genotypes (HPV6, HPV11, HPV16, HPV18, HPV31, HPV33, HPV45, HPV52, and HPV58).

The vaccines were originally licensed as a three-dose schedule, but a two-dose schedule was approved in girls younger than 15 years in 2016.^4^ However, the costs of setting up and sustaining a multi-dose HPV vaccine programme that targets young girls remain a barrier to HPV vaccine introduction.^5^ By the end of 2019, only 24% of low-income and middle-income countries (LMICs) had included HPV vaccination in their national immunisation schedules and complete series coverage is often low.^6^ Therefore, new vaccination approaches are needed if the WHO goal of cervical cancer elimination is to be met. A one-dose vaccine schedule, if effective, could simplify and reduce the costs of vaccine purchase and delivery, facilitate the sustainability of national programmes, and potentially increase uptake of vaccination.

Because of the challenges in accruing virological or disease endpoints for efficacy trials when HPV vaccination is given to girls before sexual debut, efficacy of the two-dose schedule of HPV vaccination in young girls has been assessed through immunobridging trials, and the schedule was approved on the basis of antibody data.^7^, ^8^, ^9^ In immunobridging trials, anti-HPV antibody concentrations for specific HPV genotypes in a new population group are compared with those in a population group where efficacy has been shown, with the aim of showing that immune responses in the new population are non-inferior to those seen in the original population. If immune responses are shown to be non-inferior, then efficacy is also assumed to be comparable.

Data from observational studies suggest that one dose of HPV vaccine might confer durable protection against HPV infection and cervical cancer precursors up to 11 years after vaccination.^10^, ^11^ Recently, the first randomised trial of single dose efficacy, the KEN SHE trial, in sexually active women aged 15–20 years, found 97·5% efficacy against incident persistent HPV16 and HPV18 infection at 18 months compared with a control vaccine.^12^

We did a randomised trial of reduced dose schedules of two HPV vaccines in girls aged 9–14 years in Tanzania to establish whether a single dose of HPV vaccine produces immune responses that are likely to be effective in preventing cervical cancer in sub-Saharan Africa.^13^ Here we report immunobridging results at 24 months after vaccination, one of the trial's primary objectives, comparing immune responses after one dose in girls aged 9–14 years in Tanzania with those in historical cohorts of girls and young women aged 10–25 years who received one dose and in whom efficacy has been reported.^14^, ^15^

## Methods

### Study design and population

In this open-label, randomised controlled trial (Dose Reduction Immunobridging and Safety Study [DoRIS]), we assessed the immunogenicity of two HPV vaccines, the 2-valent HPV vaccine Cervarix and 9-valent vaccine Gardasil-9, in Mwanza, in northwestern Tanzania. Trial procedures have been published previously.^16^ Briefly, girls aged 9–14 years were recruited from 54 government schools. Girls were eligible if they were healthy (as determined by a physician on the basis of medical history and a physical examination) and HIV negative. Full eligibility criteria have been published elsewhere.^13^

The trial was approved by the Tanzanian Medical Research Coordinating Committee (NIMR/HQ/R.8A/Vol.IX/2236) and the ethics committee of the London School of Hygiene & Tropical Medicine (11568). Written or thumbprint informed consent was obtained from parents or guardians of participants, with written or thumbprint assent from participants.

For our immunobridging analysis, we chose two historical cohorts that received one dose of HPV vaccine. These cohorts came from two HPV vaccine trials: the Costa Rica Vaccine trial (CVT)^14^ and the Institutional Agency for Research on Cancer (IARC) India trial.^15^ We chose these studies because they are the only two large-scale studies of one dose of HPV vaccine to our knowledge that have data on long-term efficacy (11 years for CVT and 9 years for the IARC India trial). Although the IARC India trial used the 4-valent vaccine Gardasil rather than the 9-valent vaccine, both vaccines have the same manufacturer (Merck), and have similar immunogenicity and efficacy against HPV16 and HPV18.^17^ The 9-valent vaccine contains a higher dose of antigen and adjuvant than the 4-valent vaccine: 60 μg of HPV16 and 40 μg of HPV18 L1 antigens and 500 μg aluminium hydroxyl-phosphate sulfate adjuvant compared with 40 μg of HPV16 and 20 μg of HPV18 L1 antigens and 250 μg of adjuvant, respectively.

### Randomisation and masking

Girls in DoRIS were randomly assigned (1:1:1:1:1:1), using random permuted block sizes of 12, 18, and 24, to one of six groups comprising three different dose schedules of the 2-valent HPV vaccine Cervarix or 9-valent vaccine Gardasil-9: a three-dose schedule given over 6 months; two doses given over 6 months; or a single dose. The randomisation list was computer-generated by an independent statistician and trial participant identification numbers assigned sequentially in the order of treatment allocation and put into opaque sealed envelopes. Due to the nature of the intervention, once assigned treatment allocation was open label.

### Procedures

In DoRIS, girls were asked to collect a vaginal swab before vaccination, which was used to detect HPV DNA. We collected blood samples for HPV immune responses including IgG antibodies to HPV16 and HPV18 VLPs and antibody avidity at baseline, and month 1, 7, 12, 24, and 36. Girls in the one-dose and two-dose groups have been enrolled in a trial extension and samples will also be taken at month 60. Here we report data from the 24-month follow-up visit for the one-dose groups.

The CVT was a community-based, double-blind, randomised, controlled trial of the 2-valent vaccine Cervarix in women aged 18–25 years in Costa Rica.^14^, ^18^ Between June 28, 2004, and Dec 21, 2005, 7466 women were enrolled and randomly assigned (1:1) to receive three doses of the 2-valent vaccine or a control vaccine (hepatitis A vaccine), given at baseline, and at 1 and 6 months. Women who did not attend the study clinic within the specified vaccination window did not receive the scheduled dose; therefore, 1480 (765 in the HPV vaccine group) women received only one or two doses of vaccine, mainly because of pregnancy and referral to colposcopy.^19^ Initial follow-up was for 4 years; blood samples for immunogenicity and cervical samples for HPV DNA testing were collected annually during that period. At the end of the trial, women in the HPV vaccine group were invited to participate in a long-term follow-up study and a new unvaccinated control group was recruited; participants were followed up twice a year until August, 2017. Vaccine efficacy against prevalent HPV16 and HPV18 infections at 11 years after HPV vaccination was 82·1% in the one-dose group (with two infections among 112 women), 83·8% in the two-dose group (with one infection among 62 women), and 80·2% in the three-dose group (with 27 infections among 1365 women) compared with the unvaccinated group (with 178 infections among 1783 women). There was no evidence of differences in vaccine efficacy or HPV infection rates across dose groups.^10^ HPV16 and HPV18 geometric mean concentrations (GMCs) in the one-dose group reached a plateau at 6 months after vaccination and remained stable over 11 years.^10^, ^20^

The IARC India trial was a large, multicentre, cluster-randomised controlled trial comparing the efficacy of two doses versus three doses of the 4-valent vaccine in girls and young women aged 10–18 years.^11^, ^15^ Overall, 17 729 individuals were recruited between Sept 1, 2009, and April 8, 2010, at which point trial enrolment and vaccination was suspended by the Indian Government for reasons unrelated to the study. Therefore, some participants received fewer than their allocated number of doses, and 4950 individuals received only one dose. After suspension, the trial was converted to a longitudinal cohort study by default and a group of age-matched and site-matched unvaccinated controls were recruited. Participants have been followed up annually with blood sample collection for immunogenicity from a sample of participants representing all ages of the vaccinated population and cervical sample collection for HPV DNA testing, starting 18 months after participants got married or 6 months after their first child. Follow-up is planned until 2026. Compared with the unvaccinated group (32 infections among 1260 women), vaccine efficacy against persistent HPV16 and HPV18 infection at 10 years after vaccination was 95·4% in the one-dose group (with one infection among 2135 women) and was not significantly different from vaccine efficacy in the two-dose group (93·1%; with one infection among 1452 women) and three-dose group (93·3%; with one infection among 1460 women).^11^

In this immunobridging study, we used blood samples from all girls in the one-dose groups in DoRIS who attended the 24 month visit within a window of 22–28 months after vaccination. For the CVT and IARC India trial, we took a random sample of up to 140 participants from the one dose groups in each trial; participants were eligible for the immunobridging study if they attended the 24 month visit within the same window as in DoRIS, had efficacy data available, and had sufficient serum samples from the day 0 and month 24 visits remaining for re-testing. The one-dose 2-valent vaccine group in DoRIS was compared with one-dose group of the same 2-valent vaccine in the CVT, and the one-dose 9-valent vaccine group in DoRIS was compared with one-dose group of the 4-valent vaccine in the India trial.

We measured antibodies to HPV16 and HPV18 by type-specific VLP ELISA at the Frederick National Laboratory for Cancer Research HPV Immunology Laboratory (Frederick, MD, USA).^21^ Samples for the immunobridging analyses (ie, from day 0 and month 24) from the three trials were batched (ie, processed and analysed at the same time by the same analyst) and tested together to minimise variability. Antibody concentrations greater than or equal to the lower limit of detection were prespecified to indicate seropositivity (for HPV16, ≥1·309 international units [IU] per mL; for HPV18, ≥1·109 IU/mL).

In DoRIS, we did HPV DNA genotyping at enrolment (day 0) using the Anyplex HPV28 detection assay (Seegene, Seoul**,** South Korea) at the Catalan Institute of Oncology (Barcelona, Spain). In the CVT, PCR-based HPV DNA testing at enrolment was done at the Delft Diagnostic Laboratory (Delft, Netherlands) with amplification and probe hybridisation using the SPF10 HPV DNA enzyme immunoassay system, followed by typing with the LiPA25 version 1 line detection system.^22^ HPV DNA testing at enrolment was not done in the IARC India study.

### Outcomes

The primary outcome of the DoRIS trial was to compare HPV16-specific and HPV18-specific seropositivity in participants who received one dose of vaccine with those who received two or three doses of the same vaccine, 24 months after vaccination.^13^ For this immunobridging analysis, the overall aim was to compare vaccine-induced HPV genotype-specific immune responses in DoRIS participants who received one dose of HPV vaccine with those in two historical cohorts of girls and young women who received only one dose of HPV vaccine, in whom efficacy has been reported.

The primary immunobridging objective of the DoRIS trial was to determine whether HPV16 and HPV18 antibody GMCs at 24 months in girls who received one dose in DoRIS were non-inferior to those of one-dose historical cohorts in the CVT and IARC India studies. The secondary immunobridging objective was to determine whether HPV16 and HPV18 seropositivity was non-inferior at 24 months. The 24 month timepoint was chosen for the immunobridging objectives because one dose antibody concentrations are expected to have reached plateau levels by that timepoint.^20^

### Statistical analysis

With 155 participants in each HPV-dose schedule group in DoRIS, assuming a loss to follow up of 20% over 36 months, we expected to have 130 girls in each group at the 24 month visit for the primary non-inferiority analyses. If the true GMC ratio (DoRIS *vs* comparison cohort) between groups is 1·0, with 130 participants at 24 months in each group, we had more than 90% power to show that the lower limit of the 95% CI for the GMC ratio was greater than 0·50, indicating that the one-dose schedule in girls in Tanzania did not lead to HPV16 and HPV18 antibody GMCs of 50% or lower than those of the comparison cohort in which efficacy was observed. We assumed an SD of 0·50–0·60 log10 anti-HPV concentration,^23^ and used a one-sided non-inferiority test at the 2·5% level. If the true proportion of participants who seroconvert is the same in each group, with 130 girls per group, we had more than 90% power to show that the lower limit of the 95% CI for the difference (DoRIS minus comparison cohort) was greater than –5%, indicating that seropositivity with the one-dose schedule in Tanzania was at least more than 95% of the seropositivity in the historical cohort.

The primary immunobridging analysis was in the per-protocol cohort, which included participants who received only one dose of HPV vaccine and who were HPV antibody negative (for the DoRIS *vs* CVT and the DoRIS *vs* IARC India comparisons), and HPV DNA negative (DoRIS *vs* CVT comparison) at enrolment for the specific genotype under analysis. Secondary analyses included all participants who received one dose of HPV vaccine, irrespective of baseline antibody or HPV DNA status (ie, total vaccinated cohort).

We did separate analyses to compare immune responses after one dose of the 2-valent vaccine in DoRIS with one dose of the 2-valent vaccine in the CVT, and responses after one dose of the 9-valent vaccine in DoRIS with one dose of the 4-valent vaccine in the IARC India trial. We log10-transformed HPV genotype-specific antibody concentrations for analysis. We gave antibody concentrations below the assay cutoff a value of half the cutoff before log transformation. We calculated arithmetic mean log10 antibody concentrations and 95% CIs for each group, assuming a normal distribution.

We calculated the difference in HPV genotype-specific log10 concentrations at 24 months between the two groups (DoRIS minus comparison cohort) and its 95% CI; we obtained the GMC ratio and its 95% CI by back-transformation. The antibody response was determined to be non-inferior if the lower bound for the two-sided 95% CI for the GMC ratio was above 0·50; this margin was defined a priori on the basis of that used in several previous HPV vaccine trials.^24^, ^25^

We calculated the number and proportion of girls in each group who were seropositive for HPV16-specific and HPV18-specific antibodies at 24 months. For each vaccine type and HPV genotype, we calculated the difference (DoRIS minus comparison cohort) in the proportion who were seropositive and estimated the 95% CI for the difference using the exact method of Chan and Zhang.^26^ Non-inferiority of seropositivity was concluded if the lower bound of the two-sided 95% CI for the difference was above –5%.

In a prespecified secondary analysis, we used linear regression to compare log10 concentrations between one dose of 9-valent vaccine in DoRIS and one dose of 4-valent vaccine in the IARC India trial, adjusting for age as a categorical variable. We back-transformed regression coefficients and 95% CIs to express the estimates as GMC ratios. Because there was no overlap in the age ranges between DoRIS and CVT, we did no adjustments for age. We also did a post-hoc subgroup analysis restricted to girls who were younger than 15 years at the time of vaccination for the 9-valent vaccine group in DoRIS and the 4-valent group in IARC.

We used linear regression models with a term for study group to obtain p values; p values of less than 0·05 were considered to be statistically significant.

We used SAS (version 9.1) and Stata (version 17) for all analyses. This study is registered with ClinicalTrials.gov, NCT02834637.

### Role of the funding source

The funders of this study did not have any role in the study design, data collection, data analysis, data interpretation, or writing of the report.

## Results

Between Feb 23, 2017, and Jan 6, 2018, 1002 girls were screened for eligibility, and 930 were enrolled in DoRIS and assigned to either one dose, two doses, or three doses of 2-valent vaccine, or one dose, two doses, or three doses of 9-valent vaccine (n=155 per group; full details of enrolment and randomisation have been published elsewhere^13^). 154 (99%) of 155 participants in the one-dose 2-valent vaccine group and 152 (98%) of 155 in the one-dose 9-valent vaccine group attended the 24 month visit within the 22–28 month window and so were eligible for the total vaccinated cohort for the immunobridging analysis. In the CVT, 115 (42%) of 277 one dose recipients were eligible, and all were included in the immunobridging analysis. In the IARC India trial, 139 (93%) of 150 eligible one dose recipients were randomly selected for this analysis.

Baseline characteristics were similar between the two one-dose groups in DoRIS but, because of the design of the different trials, DoRIS participants were younger than the one-dose recipients in the CVT and IARC India trial (table 1). Baseline HPV16 and HPV18 seropositivity was similar between the DoRIS and IARC India trial participants, and lower in the DoRIS trial than in the CVT, consistent with the older age range of the CVT.

**Table 1:** Demographic characteristics at baseline among one dose recipients in DoRIS included in immunobridging analyses, by vaccine received, and one dose recipients in historical cohorts

	**DoRIS (2-valent vaccine; n=154)**	**CVT (2-valent vaccine; n=115)**	**DoRIS (9-valent vaccine; n=152)**	**IARC India (4-valent vaccine; n=139)**
**Age, years**				
Median	10 (9–12)	21 (19–23)	10 (9–12)	14 (13–16)
9–14	154 (100%)	0	152 (100%)	74 (53%)
15–19	0	115 (100%)	0	65 (47%)
**HPV16 seropositive at baseline**				
Yes	6 (4%)	16 (14%)	7 (5%)	8 (6%)
No	148 (96%)	99 (86%)	145 (95%)	131 (94%)
**HPV18 seropositive at baseline**				
Yes	13 (8%)	16 (14%)	16 (11%)	9 (6%)
No	141 (92%)	99 (86%)	136 (89%)	130 (94%)
**HPV16 DNA positive at baseline**				
Yes	0	3 (3%)	1 (1%)	NA*
No	154 (100%)	112 (97%)	151 (99%)	NA*
**HPV18 DNA positive at baseline**				
Yes	0	4 (3%)	1 (1%)	NA*
No	154 (100%)	111 (97%)	151 (99%)	NA*
**HPV16 seropositive or DNA positive at baseline**				
Yes	6 (4%)	18 (16%)	7 (5%)	NA*
No	148 (96%)	97 (84%)	145 (95%)	NA*
**HPV18 seropositive or DNA positive at baseline**				
Yes	13 (8%)	18 (16%)	16 (11%)	NA*
No	141 (92%)	97 (84%)	136 (89%)	NA*
**Included in per-protocol analysis**				
HPV16	148 (96%)	97 (84%)	145 (95%)	131 (94%)
HPV18	141 (92%)	97 (84%)	136 (89%)	129 (93%)

Data are median (IQR) or n (%). CVT=Costa Rica Vaccine trial. DoRIS=Dose Reduction Immunobridging and Safety Study. HPV=human papillomavirus. IARC=Institutional Agency for Research on Cancer. NA=not applicable.

* Baseline DNA status was not measured in IARC India trial.

In the per-protocol comparison of the 2-valent vaccine, 147 (99%) of 148 participants in DoRIS and 96 (99%) of 97 participants in the CVT were seropositive for IgG antibodies to HPV16 at 24 months, and 139 (99%) of 141 in DoRIS and 96 (99%) of 97 in the CVT were seropositive for IgG antibodies for HPV18 (table 2). HPV16 and HPV18 antibody GMCs were higher after one dose of the 2-valent vaccine in DoRIS than in CVT, although the difference was not significant (table 2). Non-inferiority of antibody concentrations for the 2-valent vaccine was met for both HPV genotypes, with GMC ratios (DoRIS *vs* CVT) of 1·30 (95% CI 1·00 to 1·68) for HPV16 and 1·23 (0·95 to 1·60) for HPV18. Non-inferiority was also met for seropositivity, with a difference in seroconversion (DoRIS minus CVT) of 0·4% (95% CI –3·1 to 5·1) for HPV16 and –0·4% (–4·4 to 4·4) for HPV18 (table 3).

**Table 2:** GMCs and seroconversion rates at 24 months after a single dose HPV vaccination between DoRIS and historical cohorts (per-protocol population*)

	Participants*	**GMC (IU/mL)**†	**Seroconversion**‡
**HPV 16 IgG antibody**			
DoRIS (2-valent vaccine)	148	22·9 (19·9–26·4; 14·7–40·0)	147 (99%)
CVT (2-valent vaccine)	97	17·7 (13·9–22·5; 7·3–38·7)	96 (99%)
DoRIS (9-valent vaccine)	145	13·7 (11·9–15·8; 8·9–21·4)	144 (99%)
Aged <15 years (post hoc)	145	13·7 (11·9–15·8; 8·9–21·4)	144 (99%)
India (4-valent vaccine)	131	6·7 (5·5–8·2; 3·3–16·1)	121 (92%)
Aged <15 years (post hoc)	68	9·7 (7·7–12·1; 5·0–21·1)	68 (100%)
**HPV 18 IgG antibody**			
DoRIS (2-valent vaccine)	141	9·9 (8·5–11·5; 5·7–17·7)	139 (99%)
CVT (2-valent vaccine)	97	8·0 (6·4–10·0; 3·7–15·5)	96 (99%)
DoRIS (9-valent vaccine)	136	5·7 (4·9–6·8; 3·0–10·8)	133 (98%)
Ages 15 years (post hoc)	136	5·7 (4·9–6·8; 3·0–10·8)	133 (98%)
India (4-valent vaccine)	129	2·2 (1·9–2·7; 1·2–4·1)	99 (77%)
Ages <15 years (post hoc)	69	2·7 (2·1–3·4; 1·4–4·5)	57 (83%)

Data are n, GMC (95% CI; IQR), or n (%), unless otherwise stated. CVT=Costa Rica Vaccine trial. DoRIS=Dose Reduction Immunobridging and Safety Study. HPV=human papillomavirus. IARC=Institutional Agency for Research on Cancer. GMC=geometric mean concentration.

* Includes DoRIS and CVT participants who were ELISA antibody negative and HPV DNA negative, and IARC India participants who were ELISA antibody negative, at baseline (before vaccination) for the HPV genotype under analysis.

† ELISA serum antibody GMC.

‡ Seroconversion was defined as concentrations greater than or equal to the laboratory determined cutoff (HPV16=1·309 IU/mL; HPV18=1·109 IU/mL) among girls who were seronegative for the HPV genotype at baseline.

**Table 3:** Comparison of GMCs and seroconversion rates at 24 months after a single dose HPV vaccination between DoRIS and historical cohorts (per-protocol population*)

	**GMC ratio (DoRIS/historical cohort)**	**Adjusted GMC ratio**†	**Difference in seroconversion (DoRIS − historical control)**
**HPV16 IgG antibody**			
DoRIS *vs* CVT	1·30 (1·00 to 1·68)	..‡	0·4% (−3·1 to 5·1)
DoRIS *vs* IARC India	2·05 (1·61 to 2·61)	1·29 (0·91 to 1·82)	6·9% (2·4 to 13·1)
Aged <15 years (post hoc)	1·42 (1·10 to 1·83)	1·29 (0·94 to 1·76)	−0·7% (−4·0 to 5·0)
**HPV18 IgG antibody**			
DoRIS *vs* CVT	1·23 (0·95 to 1·60)	..‡	−0·4% (−4·4 to 4·4)
DoRIS *vs* IARC India	2·57 (2·02 to 3·27)	1·75 (1·22 to 2·50)	21·0% (13·5 to 29·5)
Aged <15 years (post hoc)	2·12 (1·59 to 2·83)	1·75 (1·23 to 2·49)	15·2% (6·1 to 26·3)

Data in parentheses are 95% CIs. CVT=Costa Rica Vaccine trial. DoRIS=Dose Reduction Immunobridging and Safety Study. HPV=human papillomavirus. IARC=Institutional Agency for Research on Cancer. GMC=geometric mean concentration.

* Includes DoRIS and CVT participants who were ELISA antibody negative and HPV DNA negative, and IARC India participants who were ELISA antibody negative, at baseline (before vaccination) for the HPV genotype under analysis.

† Adjusted for age.

‡ Adjustment not done for comparisons between DoRIS and CVT because there is no overlap in the age range.

In the per-protocol comparison of the 9-valent vaccine with the 4-valent vaccine, 144 (99%) of 145 participants in DoRIS and 121 (92%) of 131 in the IARC India trial were seropositive for IgG antibodies to HPV16 at 24 months, and 133 (98%) of 136 in DoRIS and 99 (77%) of 129 in the IARC India trial were seropositive for IgG antibodies to HPV18 (table 2). For both HPV genotypes, antibody GMCs were higher after one dose of the 9-valent vaccine in DoRIS than after one dose of the 4-valent vaccine in the IARC India trial (HPV16 and HPV18: p<0·0001). Non-inferiority of antibody concentrations was met for the 9-valent versus 4-valent vaccine for both HPV genotypes, with GMC ratios (DoRIS *vs* IARC India trial) of 2·05 (95% CI 1·61–2·61) for HPV16 and 2·57 (2·02–3·27) for HPV18. After adjusting for age, the GMC ratios were 1·29 (95% CI 0·91–1·82) for HPV16 and 1·75 (1·22–2·50) for HPV18. Non-inferiority of seropositivity at 24 months was also met, with a difference (DoRIS minus IARC India trial) of 6·9% (95% CI 2·4–13·1) for HPV16 and 21·0% (13·5–29·5) for HPV18.

In secondary analyses in the total vaccinated cohort, we found non-inferiority of antibody GMCs and seropositivity for the 2-valent vaccine (DoRIS *vs* CVT) and 9-valent versus 4-valent vaccine (DoRIS *vs* IARC India trial) comparisons for both HPV genotypes (figure; appendix 2 p 1). In the post-hoc subgroup analysis comparing one dose of the 9-valent vaccine in girls in DoRIS with the 4-valent vaccine restricted to girls younger than 15 years in the IARC India trial, we found non-inferiority of antibody GMCs and seropositivity (Table 2, Table 3).

**Figure:**
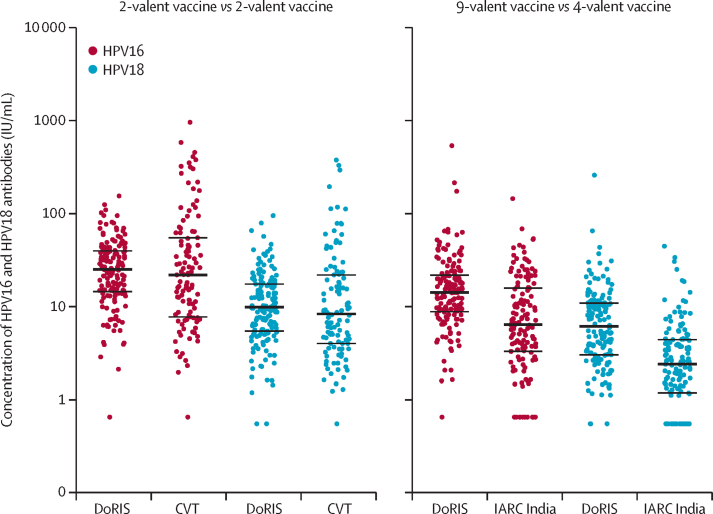
Distribution of HPV16 and HPV18 antibody concentrations at 24 months after a single dose of HPV vaccine, by study group (total vaccinated cohort) Each datapoint represents a single individual and the line through the datapoints indicates the median concentration, with IQR shown by error bars. CVT=Costa Rica Vaccine trial. DoRIS=Dose Reduction Immunobridging and Safety Study. HPV=human papillomavirus. IARC=Institutional Agency for Research on Cancer.

## Discussion

In this immunobridging study, including the first randomised trial of a single dose of HPV vaccine in girls aged 9–14 years, we found that immune responses at 24 months in girls in Tanzania were non-inferior to those in study populations aged 18–25 years in Costa Rica and 10–18 years in India who received one dose and in whom one-dose efficacy against persistent HPV infection has been reported.^14^, ^15^ These encouraging results show that a single dose of HPV vaccine induces immune responses that are comparable in different populations and geographical contexts, and add to the evidence that a single dose is likely to be effective against persistent HPV16 and HPV18 infection and associated disease.

Recently, the first randomised controlled trial of single-dose efficacy (KEN SHE), in Kenyan girls and women aged 15–20 years, found that efficacy of both the 2-valent vaccine Cervarix and the 9-valent vaccine Gardasil-9 against persistent HPV16 and HPV18 infection at 18 months after vaccination was 97·5% compared with the meningococcal vaccine control group.^12^ We are planning to do an immunobridging analysis of the DoRIS results and the KEN SHE results in the future.

In April, 2022, WHO's Strategic Advisory Group of Experts on Immunization met to assess the evidence on the efficacy of the single-dose HPV vaccination schedule, including the results from DoRIS. The committee recommended that the HPV vaccine dose schedule be updated to allow countries to choose a one-dose or two-dose schedule for girls aged 9–14 years and for young women aged 15–20 years.^27^

Because HPV-related disease (cervical intraepithelial neoplasia grade 2 or worse) and virological endpoints (persistent infection) might take a long time to accrue and require costly studies, gynaecological examinations, and sampling that might be considered unacceptable in girls in some settings, WHO recommends that immunobridging trials are appropriate for licensure of new dose schedules of HPV vaccines in young adolescents.^8^, ^28^ Although there is no defined immune correlate of protection to inform licensure, non-inferiority of antibody concentrations is recommended as the main trial endpoint. This recommendation aligns with the large body of evidence that protection after HPV L1 VLP vaccination is mediated via systemic induction of neutralising antibodies, which are effective at very low concentrations.^29^ Antibody concentrations after one dose are known to be inferior to two or three doses, despite similar efficacy. Therefore, licensure of a single dose schedule requires efficacy trials with virological endpoints, along with well-designed immunobridging studies comparing antibody concentrations after one dose in different population groups to antibody concentrations in populations in which virological efficacy of one dose has been reported. If antibody concentrations in the new population are shown to be non-inferior to those in populations in which efficacy has been found, then protection is also expected to be the same.

When comparing antibody GMCs, we used a non-inferiority margin of 0·50, which was met for all comparisons. If we had used a more stringent margin of 0·67, indicating that antibody GMCs in DoRIS were not reduced by more than 33%, it would also have been met in both the per-protocol and total vaccinated cohort analyses for each trial, and the post-hoc comparison of antibody responses among girls younger than 15 years in the IARC India trial. In the total vaccinated cohort, antibody concentrations in participants in DoRIS remained non-inferior to those of the historical cohorts; although 16% of participants in CVT were HPV16 or HPV18 DNA or seropositive at enrolment and so vaccination might have acted as a booster of their response to natural infection. Interestingly, GMCs in DoRIS were not significantly higher than those in the CVT, despite the older age of participants in the CVT than in DoRIS. The higher GMCs and seroconversion rates observed in DoRIS than in the IARC India trial might in part be due to the higher dose of antigen and adjuvant in the 9-valent vaccine than in the 4-valent vaccine, particularly for HPV18, for which the antigen dose has been doubled. This finding might also be explained in part by the age difference, because participants in DoRIS were younger on average than those in the IARC India trial.

Data from the CVT have shown that one dose of the 2-valent vaccine provides sustained HPV16 and HPV18 antibody levels for at least 11 years and that vaccine efficacy among women who received one dose was not significantly different from those who received three doses.^10^ Similarly, the IARC India trial has shown sustained antibody levels after one dose of the 4-valent vaccine with no difference in protection against persistent HPV16 and HPV18 infection compared with three doses for up to 9 years.^11^

Although participants in DoRIS were on average younger than those in the CVT and IARC India trials, restricting to the same age group in the IARC India study in a post-hoc analysis made no difference to the results. Because vaccinating girls in preadolescence produces higher GMCs than when vaccinated later in life,^23^, ^24^ the age difference is unlikely to affect results at later timepoints.

Strengths of our study include the immunobridging analysis of results for two HPV vaccines in two population groups among whom long-term efficacy has been found, allowing us to investigate the reproducibility of the one-dose results across three different geographical regions and different vaccines. DoRIS was run in a region with an extremely high burden of cervical cancer and where vaccination is most needed. We tested the samples from DoRIS, CVT, and the IARC India trial in the same batch, using a well validated assay,^21^ to minimise potential variability and allow robust comparisons between the studies.

Our study also had several limitations. One limitation of our study is that, although the vaccines used in DoRIS and the IARC India trial are similar (9-valent and 4-valent vaccines), they are not identical. However, a randomised trial of the two vaccines has shown that, despite their differences, they have similar efficacy and immunogenicity for HPV genotypes in common.^30^ Other limitations include a follow-up period of only 24 months. Immunogenicity data will also be collected from DoRIS participants at 5 years after vaccination and immunobridging analyses to later timepoints from CVT and the IARC India trial are planned. Additionally, a trial in Tanzania of one-dose HPV vaccination in boys is underway (NCT04953130).

In summary, our findings contribute to the evidence that one dose of HPV vaccine might provide strong protection against cervical cancer and be a promising strategy towards achieving cervical cancer elimination in sub-Saharan Africa and elsewhere. A single dose HPV vaccine schedule could substantially reduce the costs of vaccine purchase and delivery, alleviate vaccine supply constraints, and expand access in the countries that need it most.

## Data sharing

Deidentified participant data presented in this Article can be made available after publication following written request to the London School of Hygiene & Tropical Medicine (LSHTM) and the Mwanza Intervention Trials Unit (MITU), Tanzania. Requests must be accompanied by an analysis plan, which will be reviewed by the MITU Data Sharing Committee and lead investigators for each trial. Requesting researchers will be required to sign a Data Access Agreement if approval is given. De-identified participant data from the blinded phase of the CVT can be shared with outside collaborators for research to understand more about the performance of the HPV vaccine, immune response to the vaccine, and broader study factors associated with the natural history of HPV infection and risk factors for infection and disease. Outside collaborators can apply to access the protocols and data online; to request an application and information pack, email CVTDataSharing@westat.com. The trial summary, current publications, and contact information for the CVT are available online.

## Declaration of interests

KB, HW, and DW-J report a grant from Merck for a new study of single-dose HPV vaccination in males in Tanzania, unrelated to this submitted work. PB, ARK, JTS, HW, and DW-J are members of the Single Dose HPV Vaccine Evaluation Consortium, coordinated by PATH and funded by the Bill & Melinda Gates Foundation. PB reports a grant from GSK Biologicals for a previous study on safety and immunogenicity of Cervarix in India unrelated to this submitted work during his previous position at Chittaranjan National Cancer Institute, Kolkata, India. DW-J reports a grant from GSK Biologicals in 2007 for a previous on safety and immunogenicity of Cervarix in Tanzania, unrelated to this submitted work. JTS reports that he was a named inventor on US Government-owned HPV vaccine patents that were licensed to GlaxoSmithKline and Merck and for which the US National Cancer Institute (NCI) previously received licensing fees. NCI's licenses have now expired but JTS was previously entitled to royalties to a specified amount, as determined by federal law governing technological transfer activities by US Government employees. All other authors declare no competing interests.

## Supplementary Material

KiSwahili translation of the abstract
