# Supplementary material for: Comparing one dose of HPV vaccine in girls aged 9–14 years in Tanzania (DoRIS) with one dose of HPV vaccine in historical cohorts: an immunobridging analysis of a randomised controlled trial
Source: Lancet Glob Health. 2022 Sep 13;10(10):e1485–93. doi: 10.1016/S2214-109X(22)00306-0 (PMC9638025; doi:10.1016/S2214-109X(22)00306-0)
Supplement: KiSwahili translation of the abstract [file mmc1.pdf]

THE LANCET
Global Health

Supplementary appendix 1

This translation in KiSwahili was submitted by the authors and we reproduce it as supplied. It has not been peer reviewed. *The Lancet*'s editorial processes have only been applied to the original in English, which should serve as reference for this manuscript.

Tafsiri hii katika Kiswahili iliwasilishwa na waandishi wa makala na tunaitoa kama ilivyowasilishwa. Haijahakikiwa na wataalam wenzao. Mchakato wa kuhariri wa Lancet umetumika kwenye nakala ya awali ya kiingereza tu, ambayo inapaswa kutumika kama rejea kwa makala hii.

Supplement to: Baisley K, Kemp TJ, Kreimer AR, et al. Comparing one dose of HPV vaccine in girls aged 9–14 years in Tanzania (DoRIS) with one dose of HPV vaccine in historical cohorts: an immunobridging analysis of a randomised controlled trial. *Lancet Glob Health* 2022; **10:** e1485–93.

Kulinganisha dozi moja ya chanjo ya VIPABI kwa wasichana wenye umri wa miaka 9-14 nchini Tanzania (DoRIS) na dozi moja ya chanjo ya VIPABI kwa makundi ya kihistoria ya washiriki wa tafiti: Uchambuzi wa kulinganisha kingamwili katika utafiti uliotumia mbinu ya bahati nasibu.

Utangulizi

Chanjo dhidi ya virusi vya papilloma ya binadamu(VIPABI) zinatolewa kwa utaratibu wa dozi mbili kwa watoto wenye umri wa miaka 9- 14 au utaratibu wa dozi tatu kwa watu wazima. Tulilinganisha mwitikio wa kingamwili baada ya dozi moja ya chanjo ya VIPABI kwenye Utafiti wa Kupunguza Dozi, na kulinganisha kingamwili na Usalama wa chanjo(DoRIS), utafiti unaotumia mbinu ya bahati nasibu na taratibu tofauti za kutoa chanjo ya VIPABI nchini Tanzania, na ule kutoka tafiti mbili za kiuchunguzi za chanjo ya VIPABI ambazo zilionyesha ufanisi mkubwa wa dozi moja katika kipindi cha hadi miaka 11 dhidi ya VIPABI16 na VIPABI18 (Utafiti wa Chanjo wa Costa Rica[CVT] na Utafiti uliofanyika India, na Taasisi ya kimataifa ya Utafiti wa Saratani[IARC]).

Mbinu

Katika uchambuzi huu wa kulinganisha kingamwili kwenye utafiti wa wazi unaotumia njia ya bahati nasibu na uliodhibitiwa ("open-label randomised controlled trial"), wasichana walioshirikishwa katika utafiti wa DoRIS walitoka katika shule 54 za serikali ndani ya Mwanza, Tanzania,. Wasichana walikuwa na sifa za kushiriki kama walikuwa na umri wa miaka 9- 14, wenye afya njema na wasiokuwa na maambukizi ya VVU. Washiriki waliwekwa kwa kutumia njia ya bahati nasibu katika mpangilio wa (1:1:1:1:1:1) kwa kutumia bloku zilizoruhusiwa za ukubwa ("permutated block sizes") wa 12, 18 na 24 kwa dozi moja, mbili au tatu za chanjo ya valenti 2(Cervarix, GSK Biologicals, Rixensart, Belgium) au za chanjo ya valenti 9 (Gardasil 9, Sanofi Pasteur MSD, Lyon, France). Kwa uchambuzi huu wa kulinganisha kingamwili, lengo la msingi lilikuwa ni kulinganisha wastani wa wingi wa kinga mwili kijiometriki (GMC) katika kipindi cha miezi 24 baada ya dozi moja kwa washiriki waliofuata muongozo wa utafiti ikilinganishwa na washiriki wa tafiti za kihistoria: kundi la dozi moja ya chanjo ya valenti 2 kwenye utafiti wa DoRIS lililinganishwa na waliopokea chanjo ya valenti 2 ya Cervarix kutoka CVT na kundi la dozi moja ya chanjo ya valent 9 kwenye utafiti wa DoRIS lililinganishwa na waliopokea chanjo ya valenti 4 ya Gardasil(Merck Sharp & Dohme, Whitehouse Station, NJ, USA) kutoka kwenye utafiti wa India IARC. Sampuli zilichunguzwa pamoja kwa kutumia njia ya ELISA iliyotumia chembechembe zinazofanana na virusi("virus-like particle") za VIPABI16 na VIPABI18 katika kugundua kingamwili ya IgG. Kutokuwa duni kwa uwiano wa GMC (Utafiti wa DoRIS dhidi ya washiriki wa kihistoria wa utafiti) ulifafanuliwa awali kama kiwango cha chini cha asilimia 95 CI kilikuwa juu zaidi ya 0.50. Utafiti huu umesajiliwa kwenye ClinicalTrials.gov, NCT02834637.

Matokeo

Kati ya Februari 23, 2017, na Januari 6, 2018, wasichana wasichana wapatao 1002 walifanyiwa tathmini kuona kama wanastahili kushiriki i, kati yao 930 walisajiliwa katika uatafiti wa DoRIS na kila 155 walipangwa kwenye dozi moja, mbili au tatu za chanjo ya valenti 2 au dozi moja, mbili au tatu za chanjo ya valent 9. Washiriki 154(99%) kwenye kundi la dozi moja ya chanjo ya valenti 2 (umri wa kati wa washiriki miaka 10 [IQR 9- 12]) na 152(98%) kwenye kundi la dozi moja ya chanjo ya valenti 9(umri wa kati wa washiriki miaka 10 [IQR 9- 12]) walichanjwa na wakahudhuria hudhurio la miezi 24, na hivyo waliingizwa kwenye uchambuzi. Washiriki 115 waliopokea dozi moja kutoka CVT (umri wa kati wa washiriki miaka 21[19-23]) na 139 waliopokea dozi moja kutoka utafiti wa India IARC (umri wa kati wa washiriki miaka 14[13- 16]) waliingizwa kwenye uchambuzi. Miezi 24 baada ya kuchanjwa, wingi wa kinga mwili kijiometriki dhidi ya VIPABI16 ilikuwa 22.9 "international units"(IU) kwa mL(95% CI 19·9–26·4; n=148) kwa kundi la chanjo ya valenti 2 la DoRIS ikilinganishwa na 17.7 IU/mL(13·9–22·5;

n=97) kwa CVT (wastani wa wingi wa kinga mwili 1.30[95% CI 1.00-1.68]) na 13.7 IU/ mL(11·9–15·8; n=145) kwa kundi la chanjo ya valenti 9 la DoRIS ikilinganishwa na 6.7 IU/mL (5·5–8·2; n=131) kwenye utafiti wa India IARC (wastani wa kinga mwili kijometriki 2·05 [1·61–2·61]). Wastani wa kijiometriki wa kinga mwili dhidi ya VIPABI18 ilikuwa 9·9 IU/mL (95% CI 8·5–11·5: n=141) kwa kundi la chanjo ya valenti 2 la DoRIS dhidi ya 8·0 IU/mL (6·4–10·0; n=97) kwenye utafiti wa CVT (wastani wa kiojiometriki wa kinga mwili 1·23 [95% CI 0·95–1·60]) na 5·7 IU/mL (4·9–6·8; n=136) kwa kundi la chanjo ya valenti 9 la DoRIS dhidi ya 2·2 IU/mL (1·9–2·7; n=129) ya kwenye utafiti wa India IARC ((wastani wa kijiometriki wa kinga mwili 2·12 [1·59–2·83]); Kutokuwa duni kwa wingi wa kinga mwili kijiometriki kulifikiwa kwa kila chanjo dhidi ya VIPABI16 na VIPABI18.

Tafsiri

Dozi moja ya chanjo ya VIPABI kwa wasichana wadogo inaweza kutoa kinga ya kutosha dhidi ya maambukizi endelevu ya VIPABI. Utaratibu wa kutoa dozi moja unaweza kupunguza gharama, kurahisisha utoaji wa chanjo, na kuongeza upatikanaji wa chanjo.
