# Supplementary material for: Comparing one dose of HPV vaccine in girls aged 9–14 years in Tanzania (DoRIS) with one dose of HPV vaccine in historical cohorts: an immunobridging analysis of a randomised controlled trial
Source: Lancet Glob Health. 2022 Sep 13;10(10):e1485–93. doi: 10.1016/S2214-109X(22)00306-0 (PMC9638025; doi:10.1016/S2214-109X(22)00306-0)
Supplement: Supplementary appendix 2 [file mmc2.pdf]

THE LANCET
Global Health

Supplementary appendix 2

This appendix formed part of the original submission and has been peer reviewed. We post it as supplied by the authors.

Supplement to: Baisley K, Kemp TJ, Kreimer AR, et al. Comparing one dose of HPV vaccine in girls aged 9–14 years in Tanzania (DoRIS) with one dose of HPV vaccine in historical cohorts: an immunobridging analysis of a randomised controlled trial. *Lancet Glob Health* 2022; **10:** e1485–93.

Supplementary Table 1. Comparisons of geometric mean concentrations (GMC) and seroconversion rates at Month 24 post-single dose HPV vaccination between DoRIS and historical cohorts in whom efficacy has been shown (vaccinated population[1])

		N[1]	GMC[2] (95% CI) (IU/mL)	IQR[3]	Seropositive[4] N (%)	Seroconverted[5] N (%)
**Month 24**						
**HPV 16 IgG antibody**						
1 dose	DoRIS (2-valent)	154	22.7 (19.8 - 26.1)	14.5 - 39.8	153 (99.4%)	147 (95.5%)
1 dose	CVT (2-valent)	115	24.0 (18.5 - 31.2)	7.8 - 55.0	114 (99.1%)	98 (85.2%)
GMC ratio (DoRIS/CVT) (95% CI)				0.95 (0.72 -1.24)		
Adjusted GMC ratio[6] (95% CI)				-		
Difference in seroconversion (DoRIS – CVT) (95.0% CI)				10.2% (2.4-18.5)		
1 dose	DoRIS (9-valent)	152	14.1 (12.2 - 16.3)	8.8 - 21.8	151 (99.3%)	144 (94.7%)
1 dose	India (4-valent)	139	6.6 (5.4 - 8.0)	3.3 - 15.8	128 (92.1%)	121 (87.1%)
GMC ratio (DoRIS/India) (95% CI)				2.14 (1.68 -2.71)		
Adjusted GMC ratio[6] (95% CI)				1.43 (1.01 -2.00)		
Difference in seroconversion (DoRIS – India) (95% CI)				7.7% (0.9-15.0)		
**HPV 18 IgG antibody**						
1 dose	DoRIS (2-valent)	154	9.6 (8.3 - 11.1)	5.5 - 17.5	152 (98.7%)	139 (90.3%)
1 dose	CVT (2-valent)	115	10.0 (7.9 - 12.7)	4.0 - 21.9	114 (99.1%)	98 (85.2%)
GMC ratio (DoRIS/CVT) (95% CI)				0.96 (0.74 -1.25)		
Adjusted GMC ratio[6] (95% CI)				-		
Difference in seroconversion (DoRIS – CVT) (95.0% CI)				5.0% (-3.2-13.7)		
1 dose	DoRIS (9-valent)	152	6.0 (5.2 - 7.0)	3.0 - 10.9	149 (98.0%)	133 (87.5%)
1 dose	India (4-valent)	139	2.3 (1.9 - 2.7)	1.2 - 4.4	108 (77.7%)	99 (71.2%)
GMC ratio (DoRIS/India) (95% CI)				2.60 (2.07 -3.28)		
Adjusted GMC ratio[6] (95% CI)				1.87 (1.34 -2.62)		
Difference in seroconversion (DoRIS – India) (95% CI)				16.3% (6.1-25.8)		

[1]All participants (irrespective of ELISA antibody or HPV DNA status at baseline). [2]ELISA serum antibody geometric mean concentration (GMC). [3]Interquartile range, a measure of the variability, or spread, of the data. The lower and upper values represent the 25th and 75th percentile of the distribution, respectively (i.e. 50% of the data lie between these two values). [4]Positivity defined by the laboratory determined cut-off (HPV16 = 1.309 IU/mL; HPV18 = 1.109 IU/mL). [5]Seroconversion defined as concentrations greater than or equal to the laboratory determined cut-off among girls who were seronegative for the HPV genotype at baseline. [6]Adjusted for age. Adjustment not done for comparisons between DoRIS and CVT, because there is no overlap in the age range.
